# Usefulness of a medical interview support application for residents: A pilot study

**DOI:** 10.1371/journal.pone.0274159

**Published:** 2022-09-06

**Authors:** Ayaka Matsuoka, Toru Miike, Hirotaka Yamazaki, Masahiro Higuchi, Moe Komaki, Kota Shinada, Kento Nakayama, Ryota Sakurai, Miho Asahi, Kunimasa Yoshitake, Shogo Narumi, Mayuko Koba, Takashi Sugioka, Yuichiro Sakamoto

**Affiliations:** 1 Department of Emergency and Critical Care Medicine, Faculty of Medicine, Saga University, Saga City, Japan; 2 Community Medical Support Institute, Faculty of Medicine, Saga University, Saga City, Japan; San Giuseppe Hospital, ITALY

## Abstract

To conduct an appropriate medical interview, education and clinical experience are necessary. The usefulness of computer-based medical diagnostic support systems has been reported in medical interviewing. However, only a few reports have actually applied these systems and noted changes in the quality of the medical interview of residents. We aimed to examine how the use of a medical interview support application changes the medical interviews of residents. The study was conducted on 15 residents (with less than two years post-graduation) and ran from November 2020 to March 2021. Faculty members played the role of simulated patients in 20 cases, and the residents conducted the medical interviews. In 10 of the 20 cases, a medical interview support application was used. After the interview, the residents were asked to list up to 10 differential diseases; the interview was considered appropriate if it included the disease portrayed by the simulated patient. Furthermore, the duration of the medical interview, the number of questions asked, and changes in stress parameters were evaluated. The use of a medical interview support application increased the percentage of appropriate medical interviews. Considering the frequency, the use of a medical interview support application increased the rate of appropriate medical interviews in the rare disease group, as well as the number of questions and duration of the interviews. No stress reduction was observed. The medical interview support application may be a useful tool in identifying appropriate differential diseases during medical interviews by residents.

## Introduction

In actual clinical practice, medical interviews, physical examinations, and results, such as images, contribute 76%, 12%, and 11%, respectively, to a diagnosis [1]. Medical interviews play an important role in clinical diagnosis and treatment. However, improving this skill requires medical interview education and clinical experience that residents may not have [2].

To solve these problems, computer-based medical diagnostic support systems have been developed worldwide in recent years [3–5]. Recent studies have examined whether the use of these systems improves the diagnostic abilities of clinicians [6–9]. Furthermore, it has been reported that viewing the differential diagnosis of diagnostic decision support tools is beneficial to treatment policy decisions, even for residents [10]. However, there have been no reports on the educational effects and impact of computer-based medical diagnostic support systems on the quality of medical interviewing given by residents.

In this study, we examined whether the use of a computer-based medical diagnostic support system, a medical interview support application called Ubie [11], could help residents list an appropriate diagnosis. We further examined changes in interview time and the number of interviews as a result. In addition, heart rate variability analysis was used to examine whether the use of the medical interview support application reduced the mental stress of the residents conducting the interview.

## Materials and methods

### Definition of resident and participants

In Japan, high school graduates apply to the medical schools and are enrolled once they pass the entrance examination. After six years of medical education and training, students take the national examination for medical practitioners and obtain their medical license. The first two years after obtaining the license, the new medical doctors receive education in accordance with the initial training system. In this study, we have defined a "resident" as a physician who undergoes initial training within two years of obtaining a license. During the two-year initial training program, they rotate every one to three months through a variety of specialties, including internal medicine, surgery, pediatrics, obstetrics and gynecology, emergency medicine, and psychiatry. The order in which specialties are rotated during the two years varies from resident to resident. Residents who complete the initial training program are able to pursue advanced training in the departments associated with their field of specialization. The participants of this study were residents who were enrolled in Saga University Hospital in 2020 and 2021. The 15 residents who gave their informed consent participated in this study. The information of the simulated patients was based on real patient information. The patient information was completely anonymized. In addition, an opt-out opportunity was provided for all patients whose information might be used. The data collection was done between November 2020 and March 2021. A total of 300 interviews were conducted to analyze the impact of the medical interview application on the residents’ interviews.

### Method for selecting diseases for simulated patients

We divided the patients into two groups (high- and low-frequency disease groups) based on the final diagnoses of those who were transported to our emergency center between April 2015 and March 2020. Diagnosis Procedure Combination (DPC) data were used to extract the name of patients’ diseases [12]. From April 2015 to March 2020, 34379 patients were admitted to the emergency department. After excluding cases that did not lead to a definitive diagnosis, had undergone trauma, and patients with severely impaired consciousness or cardiopulmonary arrest who had difficulty in being medically interviewed, 6659 patients remained with 798 disease names. These diseases were arranged in order of the number of patients seen, and the disease group that could cover about 80% of the patients was defined as the common group (264 diseases), and the remaining were defined as the rare group (534 diseases).

**Fig 1:**
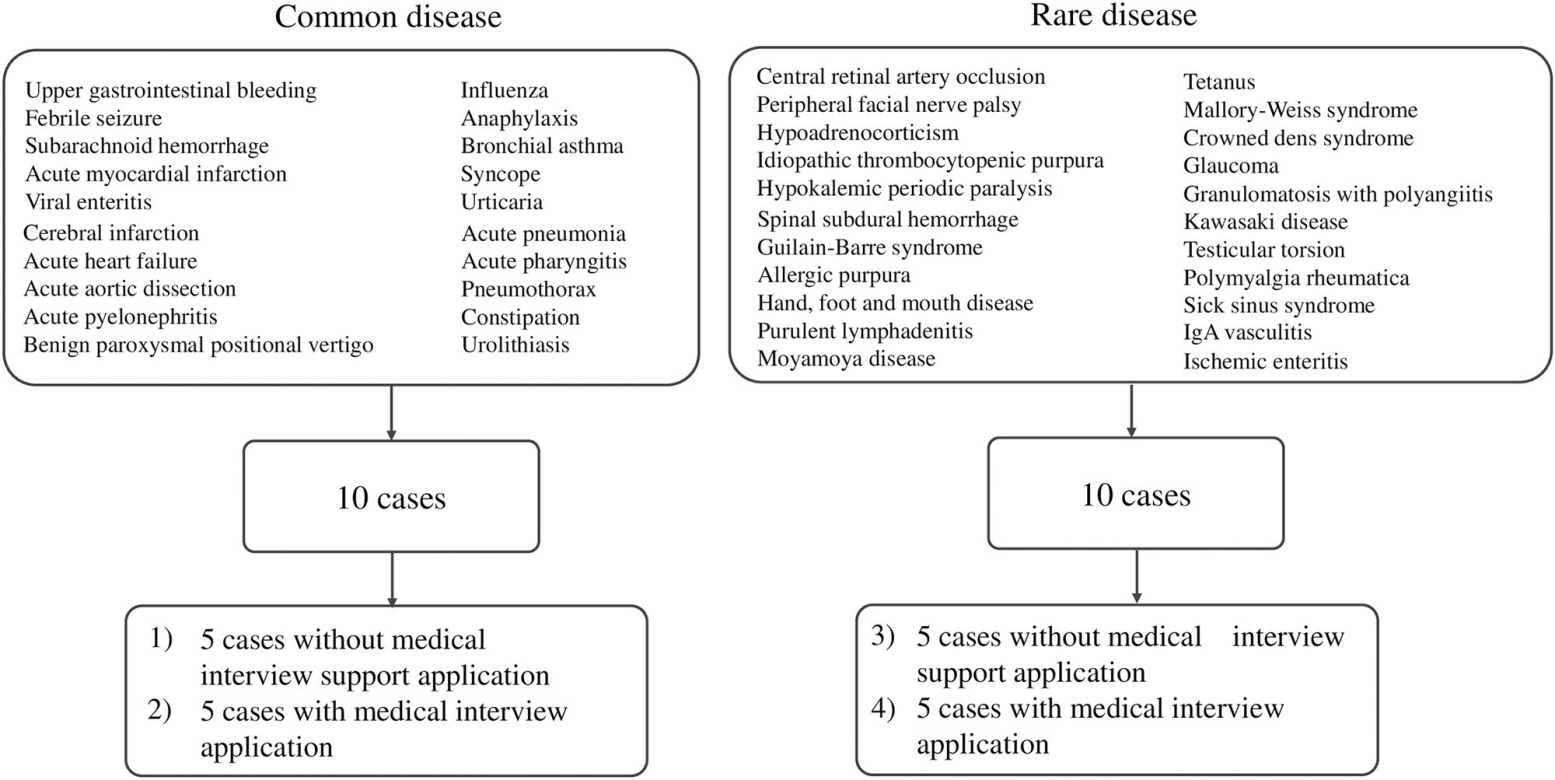
Methods of selecting the simulated patients. Ten diseases were randomly selected from each of the common and rare diseases. In each disease group, they were randomly selected and divided into two groups: one using the medical interview support application and the other not using it. The interviews were conducted in the order of (1), (2), (3), and (4).

About 20 and 22 diseases were non-randomly selected from the common (high frequency) and rare (low frequency) groups, respectively. These diseases consisted of those with a high frequency of visits to medical institutions, those with a poor prognosis due to delayed diagnosis, and those with a high fatality rate (Fig 1).

### Intervention

For the 20 randomly selected cases, faculty members acted as simulated patients and residents conducted medical interviews. Each resident was required to list, at most, 10 differential diagnoses after the interview. If the disease portrayed by the simulated patient was in the differential diagnosis, it was considered an appropriate medical interview. The number of questions, duration of the medical interview, and stress levels before and after the medical interview were measured in all medical interviews with the simulated patients. After the medical interview, the participants reported their impressions of the medical interview support application in a questionnaire survey. The primary objective of this study was to evaluate the change in the percentage of appropriate medical interviews with and without the medical interview support application. The secondary objectives were to observe the duration of the medical interview, number of questions asked, and changes in stress parameters.

### Medical interview methods

Two researchers, faculty members, who have been working in the emergency center played the roles of simulated patient and observer. The researcher who played the role of simulated patient faithfully responded to the medical interview by reviewing the contents of the extracted patients’ medical records. The researcher in the observer role recorded the duration of the interview and the number of interviews for each disease. From each of the selected disease groups (common and rare), 10 diseases were randomly selected using a random number table, and one resident conducted medical interviews for a total of 20 diseases. For the 10 diseases in each of the selected groups, 5 each were interviewed with and without the assistance of medical interview support application. When interviewing with the application, the residents conducted the interview presented by the application. However, if the resident judged that application-directed question was not necessary, that question could be skipped; furthermore, the residents were allowed to conduct an additional interview on their own.

### Medical interview support application

The medical interview support application used in this study is a question-type flowchart application based on medical dictionaries and research papers. In accordance with the flowchart, relevant questions based on the user’s answers are asked repeatedly by the program to present a list of relevant diseases. The questions are selected based on their relevance to the candidate disease. Therefore, as the medical interview proceeded, the results were narrowed to highly relevant diseases [11].

### Stress assessment methods

The stress experienced by the medical staff during the medical interview was assessed by monitoring the heart rate variability before and after the medical interview. The low-frequency (LF: frequency range 0.04–0.15 Hz) and high-frequency power (HF: frequency range 0.15–0.4 Hz) obtained from heart rate variability are influenced by sympathetic and parasympathetic nerves, respectively. The LF/HF power ratio reflects the state of the autonomic nervous system and is an indicator of mental stress [13]. By measuring the LF/HF power ratio before and after conducting a medical interview for a simulated patient, we evaluated the stress caused by conducting a medical interview and assessed whether the medical interview support application had a stress-reducing effect.

### Statistical analyses

All statistical analyses were performed using the JMP Pro version 14 software (SAS Inc., USA). Mean and median values were compared using Student’s t-test and Wilcoxon rank-sum test. The normality and distribution of continuous variables were checked using the Shapiro–Wilk test. Normally distributed data were presented as mean values, and non-normally distributed data were presented as median values. The corresponding two-group test was performed using the Wilcoxon signed-rank test. In all analyses, statistical significance was set at p < 0.05.

This study was approved by the Ethics Committee of Saga University Hospital (2020-07-R-01). Written informed consent was obtained from all study participants. We obtained opt-out consent for the actual patients from whom the simulated patient information originated. The Ethics Committee of Saga University Hospital approved this opt-out consent method.

## Results

### Primary outcome

**Fig 2:**
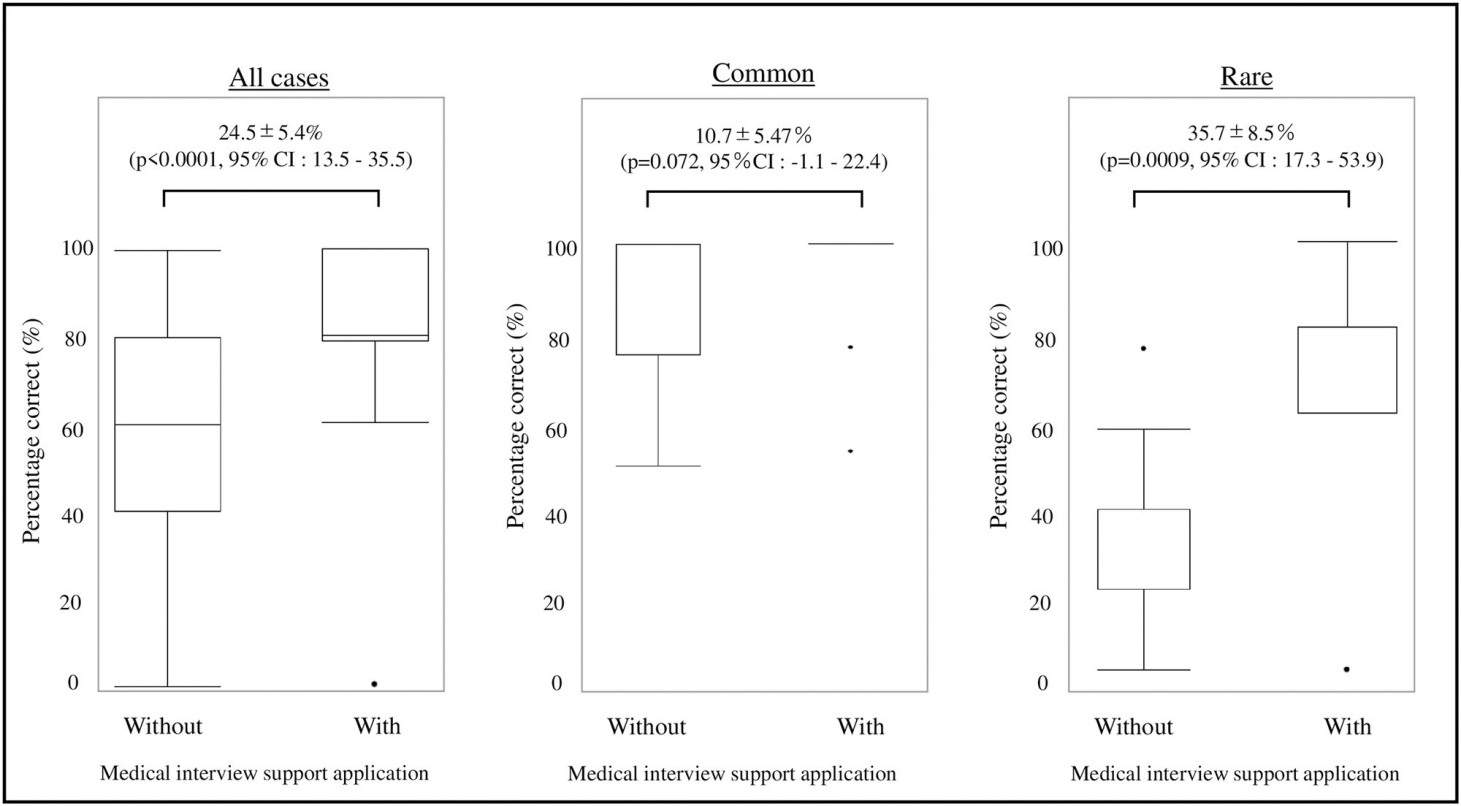
Comparison of correct answer percentages. Comparison of all cases revealed that the group that used the interview support application showed a significantly higher rate of correct answers. When validated by the frequency of diseases, the use of the interview support application increased the rate of correct answers in the rare disease group.

The difference between the percentage of appropriate medical interviews conducted with and without the use of the medical interview support application was 24.5±5.4% (p < 0.0001, 95% confidence interval [CI]:13.5–35.5) (Fig 2). Concerning frequency in the common group, the difference in the percentage for conducting an appropriate medical interview with and without the application was 10.7±5.47%, which was not significant (p = 0.072, 95% CI: -1.1–22.4). However, in the rare group, the difference increased significantly to 35.7±8.5% (p = 0.0009, 95% CI: 17.3–53.9) (Fig 2).

### Secondary outcomes

**Fig 3:**
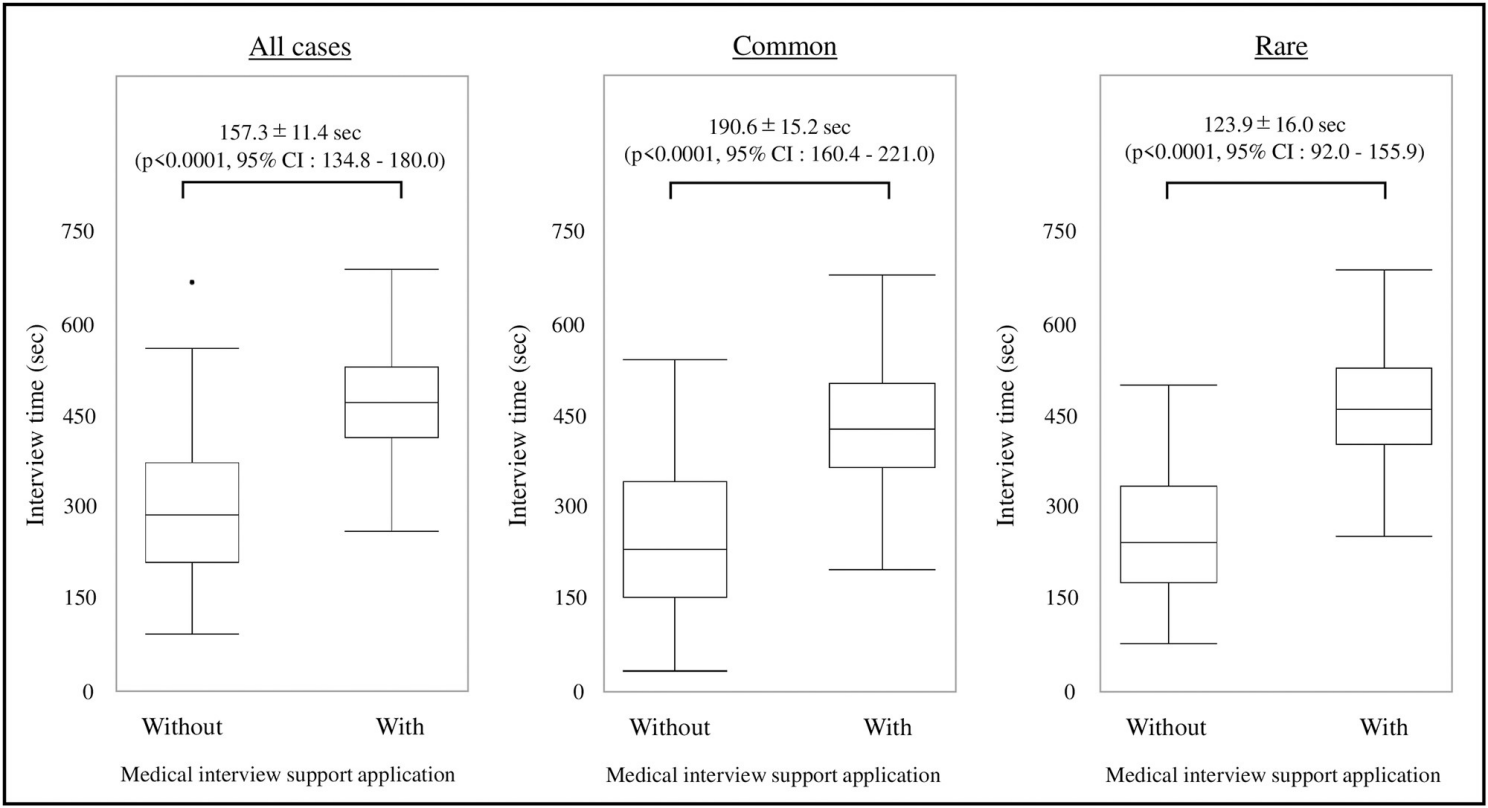
Duration of the medical interview. A box-and-whisker diagram of the interview time for the groups with and without the medical interview support application is shown. In all validations, the interview time was significantly longer for the group that used the medical interview support application.

Fig 3 shows the time required for the medical interviews. The difference in duration of time for the medical interview between the groups using and not using the medical interview support application was 157.3±11.4 seconds, which was significantly longer in the group using the application (p < 0.0001, 95% CI: 134.8–180.0). Concerning the frequency in the common group, the difference in duration of time between the application-using and non-application-using groups increased significantly to 190.6±15.2 seconds (p < 0.0001, 95% CI: 160.4–221.0). In the rare disease group, it also increased significantly to 123.9±16.0 seconds (p < 0.0001, 95% CI: 92.0–155.9).

**Fig 4:**
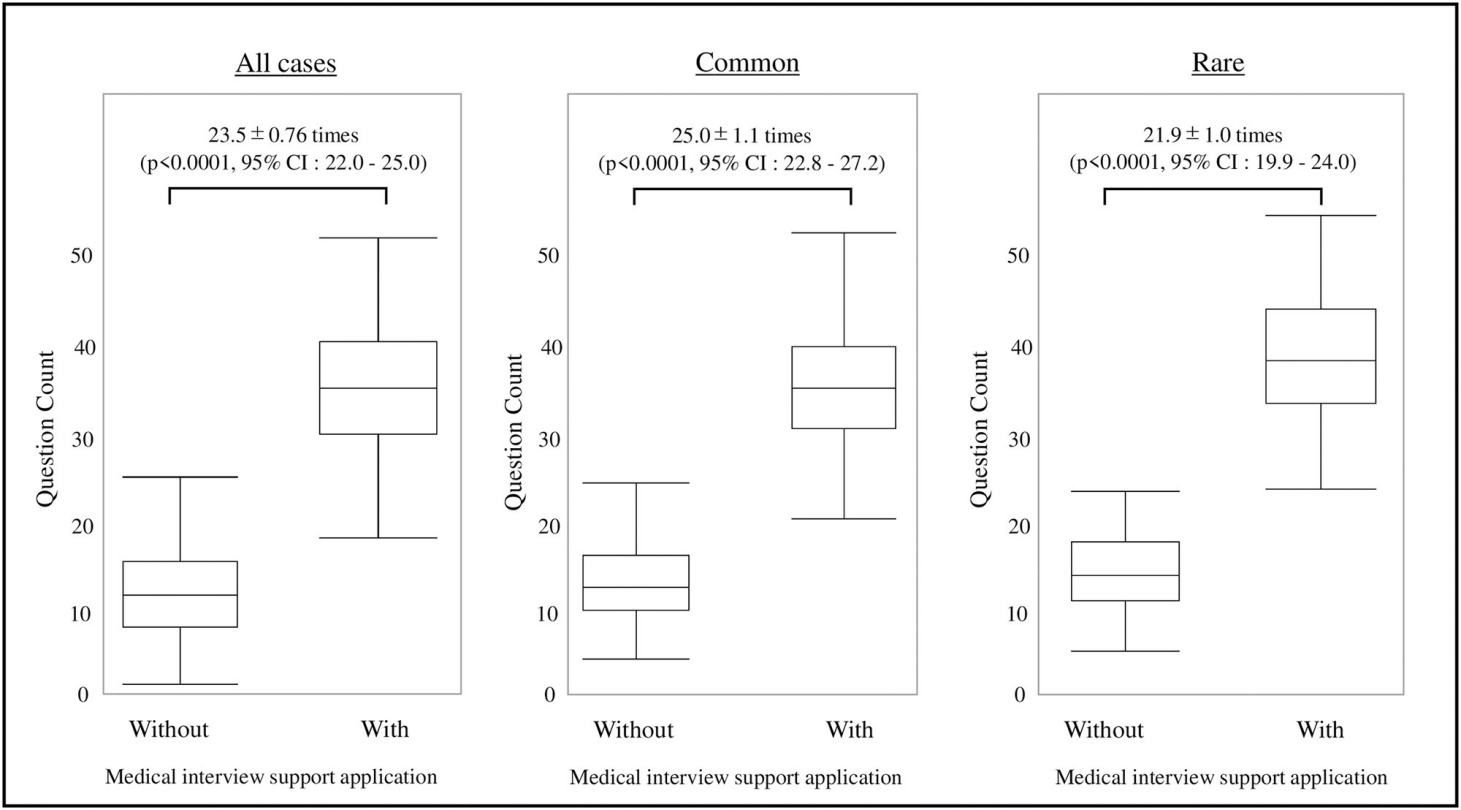
Number of questions per medical interview. A box-and-whisker diagram is shown for the number of interviews in the groups with and without the medical interview support application. In all validations, the number of interviews significantly increased in the group that used the medical interview support application.

Fig 4 shows the number of questions asked during the medical interviews. The difference in the number of questions asked between the application-using and non-application-using group was 23.5±0.76, a significant increase (p < 0.0001, 95% CI: 22.0–25.0). Concerning the frequency in the common group, the difference in the number of questions asked between the application-using and non-application-using groups increased significantly to 25.0±1.1 (p < 0.0001, 95% CI: 22.8–27.2); in the rare group, the difference increased significantly to 21.9±1.0 (p < 0.0001, 95% CI: 19.9–24.0).

**Fig 5:**
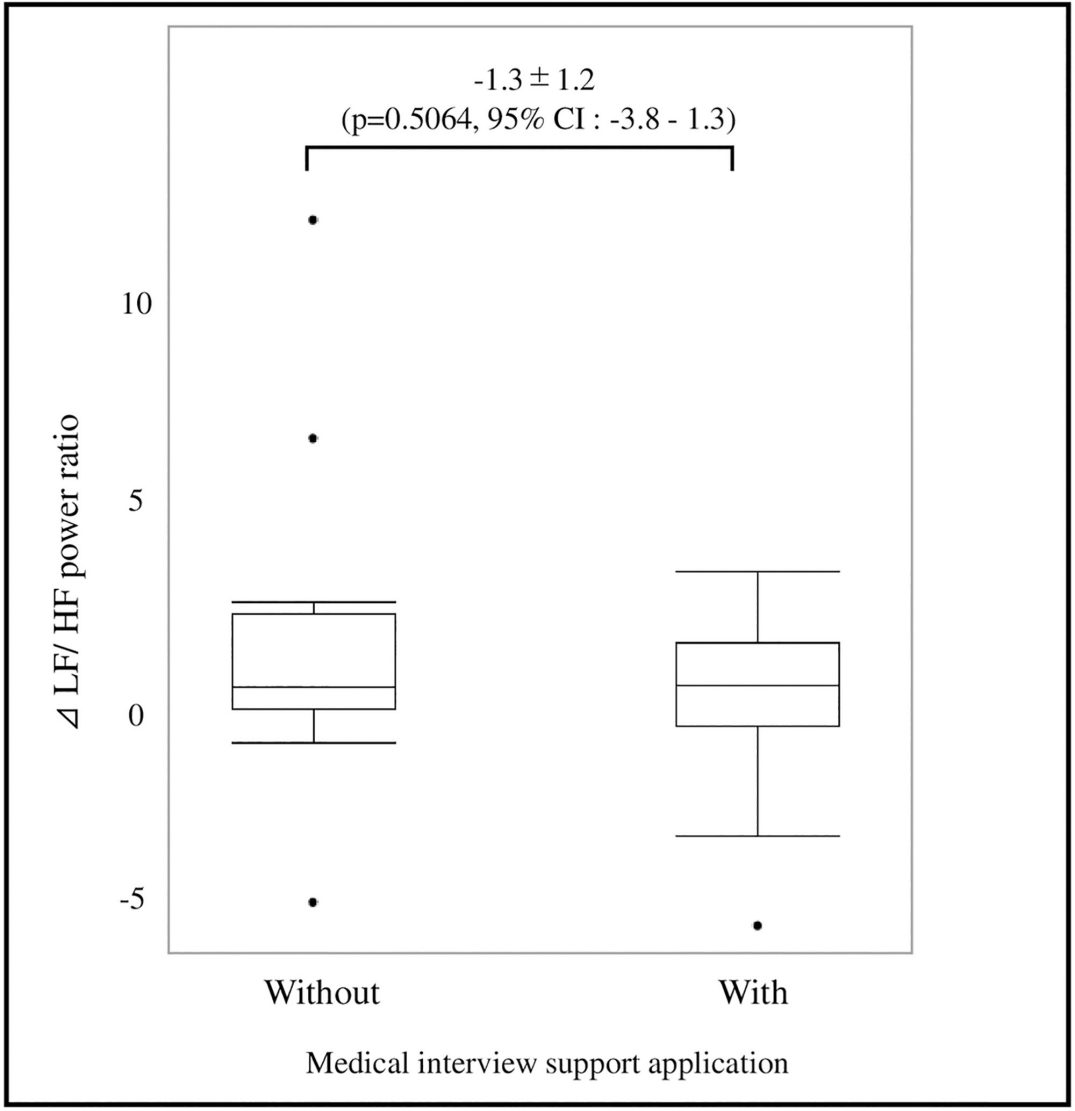
Change in stress scores by medical interview. The change in stress level before and after the interview with and without the interview support application is shown as the ⊿LF/HF power ratio. The use of the interview support application was not effective in reducing stress.

The results obtained by heart rate variability analysis are shown in Fig 5. The difference in ⊿LF/HF power ratio between the group using the medical interview support application and the group not using the application was (-1.3±1.2), and there was no effect of stress reduction by using the application (p = 0.61, 95% CI:-3.8–1.3).

In the questionnaire survey, 86.7% of the residents reported that the application would help them miss fewer necessary questions and 80% reported that it would help them identify differential diseases. Meanwhile, 42.8% of the residents stated in the open-ended questionnaire that the application made it more difficult to proceed with the interview.

## Discussion

In our study, the use of a medical interview support application significantly increased the percentage of appropriate medical interviews. Concerning the usefulness, the rare disease group benefited from the medical interview support application, while the common group did not benefit. Additionally, the use of the medical interview application extended the number and duration of interviews for the residents.

It is considered difficult for doctors to make a correct diagnosis based on the results of interviews and tests [14], and diagnostic errors are the most common cause of medical malpractice [15]. It has also been pointed out that diagnostic errors can lead to increased medical costs [16]. In these situations, the use of computer-based medical diagnostic support systems in medical interviews can help doctors obtain crucial information [17] and make a correct diagnosis [18]. In this study, when the medical interview support application was used, the duration and the number of interviews of the residents increased significantly, suggesting that they obtained more information than when the application was not used.

In the application of computer-based medical diagnostic support system in medical practice, it has been reported that it complements the physician’s diagnosis, but does not fully represent the physician’s work [14, 19]. It is also important to recognize the possibility that the correct diagnosis may not be listed in the system that is being used [20]. In this study, when the residents used the application to conduct medical interviews, they confirmed the diagnoses listed in the application, and the final list of differential diseases follows the residents’ own ideas. The results showed that the residents were able to enumerate the correct differential diagnosis using the application, which may be the result of the combination of their own thinking and the application’s effectiveness.

The computer-based medical diagnostic support systems have been investigated for their usefulness not only in clinical settings, but also in medical education [11, 21, 22]. Friedman et al. reported that residents’ use of a medical interview support application helped them identify appropriate differential diagnoses [10], which is consistent with our findings. However, it is important to note that the medical interview application was found to be useful in identifying the correct differential diseases, especially in the rare disease groups. Since the residents had limited experience with rare diseases, the medical interview application may have been effective in listing the appropriate differential diseases. Comparing the interviews conducted independently by the resident and those conducted using the application, and adding the faculty’s feedback, will broaden the residents’ perspective with respect to differential diseases, and therefore, medical interview support application could be an effective educational tool.

In the heart rate variability analysis, no stress-reducing effect was found due to use of the medical interview support application. This may reflect the fact that the residents’ interview was not completely dependent on the application. Berner et al. state that when diagnostic decision support systems are used to educate inexperienced physicians, clinical experience and knowledge are necessary to accurately evaluate the information obtained by using the system. They also point out that the interviewer must determine whether the information presented by the system is appropriate for the case, and that it can be difficult for inexperienced physicians to discern the information [23]. In the questionnaire after the medical interview, many residents said that the use of the medical interview support application reduced the possibility of failing to ask questions necessary for diagnosis. However, it was also acknowledged that it was difficult to interview patients smoothly since they were not able to perform interviews based on their own expected diseases. From this, it can be inferred that when the questions given in the application are related to diseases that the resident did not expect, the resident’s thinking is diversified because there are more differential diseases to consider. Diversified thinking through the use of applications is expected to improve the interviewing skills of the residents.

There are several limitations to this study. The samples used in this study were simulated patients based on medical record data; this is different from the actual patients. Therefore, it does not prove the effectiveness of the medical interview support application used in this study in actual clinical practice, however, it shows its effectiveness as a possibility. Additionally, the number of medical interviewers and simulated patients was small, which may have caused a selection bias.

This study demonstrated the usefulness of the medical interview support application both clinically and educationally, especially while encountering rare diseases. We are planning on expanding this pilot study by selecting a larger sample and examining how this tool affects the medical training system.

## Conclusion

Medical interview support applications can be a useful tool in identifying appropriate differential diagnoses, and the experience in the diagnostic process may have educational benefits.

## Supporting information

S1 File (DOCX)

S2 File (DOCX)
