# Supplementary material for: Usefulness of a medical interview support application for residents: A pilot study
Source: PLoS One. 2022 Sep 6;17(9):e0274159. doi: 10.1371/journal.pone.0274159 (PMC9447879; doi:10.1371/journal.pone.0274159)
Supplement: S1 File — (DOCX) [file pone.0274159.s001.docx]

**Questionnaire after using the interview application**

*1. Do you think that you miss fewer questions to patients by using the interview application?*

(1) Yes

(2) No

*2. If you answered "Yes" to 1. above, please select the items that you think are less likely to be missed. (You can select more than one)*

・The type of onset of symptoms (e.g., sudden onset or gradual exacerbation)

・The nature and severity of the symptoms (pain when pressed, etc.)

・Nature and severity of symptoms (e.g., type of pain, greatest pain in life)

・Location and dissemination of symptoms (localized or widespread)

・Accompanying symptoms (e.g., pain and fever)

・The time course of the symptom (Pain that appeared yesterday is worsening today, etc.)

*3. Do you think that the interview application will help you to identify differential diseases?*

(1) Yes

(2) No

*4. If you have any comments about the medical interview application, please feel free to write them.*
