# Supplementary material for: Usefulness of a medical interview support application for residents: A pilot study
Source: PLoS One. 2022 Sep 6;17(9):e0274159. doi: 10.1371/journal.pone.0274159 (PMC9447879; doi:10.1371/journal.pone.0274159)
Supplement: S2 File — (DOCX) [file pone.0274159.s002.docx]

**問診アプリケーション利用後アンケート**

1. 問診アプリケーションを利用することで患者さんに対して聞き逃しが少なくなると思いますか。

①はい

②いいえ

2. 1.で（はい）と答えた方は、聞き逃しが少なくなったと思われる項目を選択してください。（複数選択可能です）

・症状の発症形式（突然発症か、徐々に増悪したか）

・症状の軽快・増悪因子（押さえると痛い、など）

・症状の性質と程度（痛みの種類や、人生最大の痛みであるなど）
・症状の部位と放散（限局しているか、全体に広がっているか）

・症状の随伴症状（痛みと発熱など）

・症状の時間的経過（昨日より出現して増悪している、など）

3. 問診アプリケーションを利用することで鑑別疾患を挙げる手助けになると思いますか。

①はい

②いいえ

4. 問診アプリケーションについてご意見があれば自由に記載してください。
